# Editorial Note: Subglacial Lake Vostok (Antarctica) Accretion Ice contains a diverse set of sequences from aquatic, marine and sediment-inhabiting Bacteria and Eukarya

**DOI:** 10.1371/journal.pone.0351606

**Published:** 2026-06-15

**Authors:** The PLOS One Editors

After this article [1] was published, concerns were raised about similarities to a previously published article [2], by the same research group, which was submitted after [1] and declared prior to its publication. Specifically, [1] and [2] present metagenomic analyses of the same two ice cores.

Before the publication of [1], the corresponding author stated that [1] and [2] use the same dataset but differ in focus, analyses and conclusions. They stated that in [2], the focus is on the potential ecological conditions in Lake Vostok, while in [1], the focus is on the species and physiological types that may be present in Lake Vostok based on sequence analyses. They also stated that in [1], the sequences are 97–100% identical to sequences in the NCBI/GenBank database, and there is a more extensive analysis of the mRNA sequences in order to deduce the types of metabolic processes that might be present among organisms living in the lake.

An independent member of the *PLOS One* Editorial Board reviewed [1] and [2] and stated that the data underlying both articles are the same, with the difference that in [1], the applied similarity cut-offs for the sequencing data were ≥ 97 and 99%. They also stated that, based on the analytical pipeline descriptions in both articles, the same analysis of raw data as well as higher level analyses were performed for both articles. The *PLOS One* Editorial Board member also noted that the pre-publication statement by the authors that [1] focuses on the species and physiologies present in Lake Vostok and [2] focuses on the ecological lake environment applies to the discussion, but not the analyses performed and results presented. They therefore concluded that, based on the results presented, the analytical objectives of [1] and [2] are not distinct and do not contribute separate ecological insight.

During editorial follow-up, the corresponding author stated that the results presented in both articles were initially all included in [1], and material was removed during its revision process, further analyzed and revised, and submitted as a separate article [2].

The corresponding author also provided further information on the availability of the sequencing data, which are available on NCBI under numbers SRX326755 and SRX326747. They also clarified that the 20 m separation between the ice cores refers to their vertical depth within the glacier, which corresponds to approximately 8–10 km across the lake surface. They stated that the two samples represent distinct portions of the lake: V5 is from a shallow bay showing signs of hydrothermal activity, and V6 is from the main lake basin. The corresponding author stated that only two ice cores were available for sequencing due to the rarity of such samples.

The *PLOS One* Editors issue this Editorial Note to make readers aware of the above information.

PLOS regrets that the issues were not addressed prior to the publication of the article [1].
